# Performance Evaluation of Different Coating Materials in Delamination for Micro-Milling Applications on High-Speed Steel Substrate

**DOI:** 10.3390/mi13081277

**Published:** 2022-08-08

**Authors:** Sandeep Bhoi, Ashwani Kumar, Arbind Prasad, Chandan Swaroop Meena, Rudra Bubai Sarkar, Bidyanand Mahto, Aritra Ghosh

**Affiliations:** 1 Department of Mechanical Engineering, Parala Maharaja Engineering College, Berhampur 761003, Odisha, India; 2 Technical Education Department Uttar Pradesh, Kanpur 208024, Uttar Pradesh, India; 3 Department of Mechanical Engineering, Katihar Engineering College (Under Department of Science & Technology, Government of Bihar), Katihar 854109, Bihar, India; 4 CSIR-Central Building Research Institute, Roorkee 247667, Uttarakhand, India; 5 Research and Development Division, Tata Steel Ltd., Burma Mines, Jamshedpur 831007, Jharkhand, India; 6 Government Engineering College Vaishali (Under Department of Science & Technology, Government of Bihar), Vailshali 844115, Bihar, India; 7 College of Engineering, Mathematics and Physical Sciences, Renewable Energy, University of Exeter, Penryn, Cornwall TR109FE, UK

**Keywords:** micro-milling, wear, tool failure, coating, substrate, delamination

## Abstract

The objective of the present work is to carry out analytical and finite element analysis for commonly used coating materials for micro-milling applications on high-speed steel substrate and evaluate the effects of different parameters. Four different coating materials were selected for micro-milling applications: titanium nitride (TiN), diamond-like carbon (DLC), aluminium titanium nitride (AlTiN) and titanium silicon nitride (TiSiN). A 3D finite element model of coating and substrate assembly was developed in Abaqus to find the Hertzian normal stress when subjected to normal load of 4 N, applied with the help of a rigid ball. The radius of the rigid ball was 200 µm. For all the coating materials, the length was 3 mm, the width was 1 mm, and the thickness was 3 µm. For the high-speed steel substrate, the length was 3 mm, the width was 1 mm, and the thickness was 50 µm. Along the length and width, coating and substrate both were divided into 26 equal parts. The deformation behaviour of all the coating materials was considered as linear–elastic and that of the substrate was characterized as elastic–plastic. The maximum normal stress developed in the FEA model was 12,109 MPa. The variation of the FEA result from the analytical result (i.e., 12,435.97 MPa is 2.63%) which is acceptable. This confirms that the FEA model of coating–substrate assembly is acceptable. The results shows that the TiSiN coating shows least plastic equivalent strain in the substrate, which serves the purpose of protecting the substrate from plastic deformation and the TiSiN of 3 micron thickness is the most optimum coating thickness for micro-milling applications.

## 1. Introduction

Micro-manufacturing processes have been used extensively in the aerospace, biomedical and defense industries. Presently, photolithography-based manufacturing techniques are used for selective materials such as Ni, Cu, Si and polymers to produce high aspect-ratio components. Micro-machining processes like micro-milling are able to generate three-dimensional surfaces in ceramics, metals and polymers [1,2,3,4,5,6]. Micro-machining is becoming popular because of its ability to produce three-dimensional parts of different sizes varying from a few micrometers to a few millimeters across various materials [7]. The micro-milling, micro-EDM, micro-grinding and micro-grooving processes are part of the micro-machining process.

**Figure 1:**
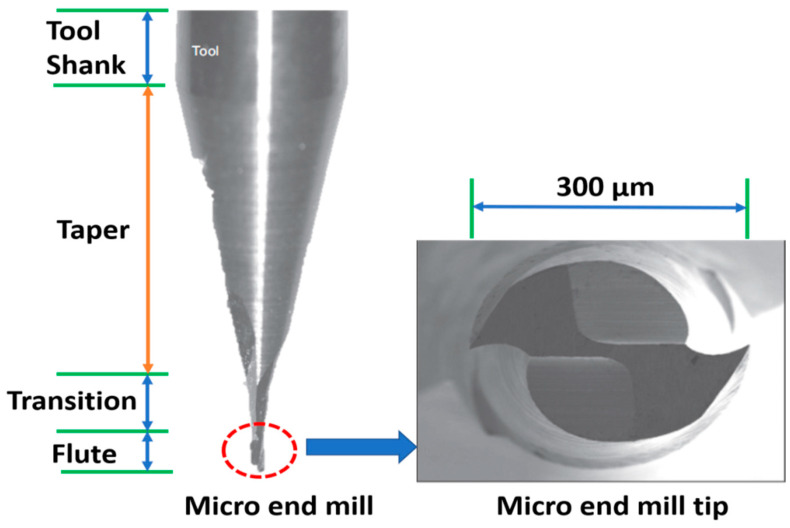
Micro-end mill and cross-section of two-flute micro-end mill [11].

Micro-milling is a micro-cutting process which is used for the fabrication of micro-and meso-scale components and devices. It can also be said that it is a milling operation at the micro-scale. However, there are vital drawbacks of the micro-milling process, particularly when machining hard materials having a hardness of 7.25–8.43 GPa and sintered ceramics having a hardness of 12.75–14.71 GPa. These drawbacks are due to the small size of the cutting tools, low flexural stiffness and strength compared to conventional-scale tools due to low material removal rates, rapid tool wear/failure and poor part feature accuracy, especially when cutting hard materials occurs [8,9,10]. Figure 1 shows a two-flute micro-end mill cutting tool with important dimensions [11]. The low flexural stiffness of these tools results in catastrophic failure because of the bending stresses generated by the cutting forces.

To prevent tool failure, a few methods have been proposed in previous research work. The first method is using cutting fluids to dissipate heat and provide lubrication. This facilitates the minimization of friction at the tool–workpiece interface and, in turn, reduces tool wear. However, this method is not very successful in micro-milling machining. The main reason is that the application of the cutting fluid to the cutting zone and the tool–workpiece interface is very difficult because of high cutting speeds and the small size of the contact zone [12]. The second method is the use of coatings on the micro-tool to reduce wear. This enhances tool life [1]. However, very little has been reported in tool-wear studies of coated micro-tools. The aim of the present work is to study the wear of micro-machining. Therefore, later in the introduction section, a brief description of wear is presented. There are several types of wear phenomena occurring in the field of mechanics, such as adhesive, abrasive, fatigue, corrosive and fretting wear as well as erosive wear by solid particles, fluids and cavitation and electric arc–induced and delamination wear. Wear of non-lubricated metal pairs sliding in a dusty environment may be termed as dry sliding wear and abrasive wear. The classification of wear processes is based on the type of wearing contacts, such as single-phase and multiple-phase.

In micro-machining, smaller tools are used. Researchers have reported that low flexural stiffness and strength causes huge bending of the tool, hampering the cutting process and leading to tool failure [5,13,14]. This is avoided by minimizing the cutting forces below a certain critical value in order to ensure that the uncut chip thickness remains sufficiently small. During the machining of steel, titanium, nickel alloys, etc. the maximum permissible chip thickness is on the order of or less than the cutting-edge radius [14,15].

In the conventional milling process, the work pieces act as isotropic and homogeneous materials, whereas in micro-milling, the smaller grains in the work piece are comparable to the size of the tool. In view of the above, the micro-milling process is very complicated [14]. The chips, which adhere to the tool, block the path at the cutting zone, and this results in an increase in the cutting forces and leads to a catastrophic failure of the tool because of its low flexural stiffness. Moreover, the small size of the micro-milling cutters makes the tip very weak because of its low stiffness value. Diamond-coated cutters are promising because of their ability to improve tool stiffness and tool life [16].

In micro-machining, the uncut chip thickness (h) is less than the cutting-edge radius (re) due to the negative effective rake angle (−α) influencing the ploughing effect in the work piece [5]. Therefore, the ratio of uncut chip thickness to cutting-edge radius is an important parameter in micro-machining [5,8]. A sharper cutting edge is required to remove the smallest amount of undeformed chip thickness [17].

The major limitations of the micro-milling process are unpredictable tool life and premature tool failure, deterioration of the cutting edge and tool wear leading to high friction generation [18,19,20,21,22,23,24,25,26,27,28,29]. It has been reported that coated micro-cutting tools have longer tool life and improved cutting performance [25]. Many researchers have used TiAlN-coated micro tools in cutting tests [19,20,23,24,28,30]. It has also been reported that CrTiAlN-coated micro-end mills provide great advantage in tool wear reduction and smooth surface finish [31,32]. The small size of micro-milling tools makes coating deposition difficult around the cutting edge. The desirable properties of the coatings for micro-machining tools are high hardness, toughness, chemical/erosive and abrasive wear resistance as well as dense and fine microstructure. Coating also provides smooth machined surfaces with a reduced coefficient of friction compared with uncoated tools [33].

TiN, TiCN, TiAlN and Al_2_O_3_ are coatings that have been frequently used in industry. Earlier studies reveal that an increase in tool life is due to an increase in hardness, greater bonding energy of the coating elements and lower friction coefficients. Due to oxidation resistance and wear resistance at higher temperatures, TiAlN coating improved cutting performance. These properties make TiAlN an appropriate coating for cutting abrasive work pieces at high speeds [34]. It has been found that the coating on a micro-milling cutting tool fails due to delamination, which was confirmed by the SEM images and EDS spectra of the worn tools [35]. Delamination wear occurs due to surface layer deformation, crack nucleation and propagation of cracks parallel to the surface. Cracks finally shear the surface, resulting in long and thin wear sheets.

Earlier it was reported in the literature through the four-point bending test that thick coatings, usually more than 2 µm, delaminate from the surface of the substrate because of their high summary toughness. Along the interface of the coating and substrate, there is a difference in material properties which facilitates delamination. Finally, the coating fails due to the buckling and spalling of the delaminated portion [36].

## 2. Research Objective

From the literature review, it was found that delamination in the coating of micro-milling cutting tools is confirmed only through SEM images and EDS spectra. However, coating material which is suitable for micro-milling applications and of appropriate thickness has not been reported in the literature. The delamination of coating from the substrate from the mechanics point of view has not been reported in the literature. Moreover, the factors on which delamination depend need to be examined. It was also found in the literature review that the coatings which are commonly used for micro-milling applications are titanium nitride (TiN), diamond-like carbon (DLC), aluminium titanium nitride (AlTiN) and titanium silicon nitride (TiSiN). It has been reported that the thickness of these coatings usually ranges from 2 to 4 microns [37,38,39,40,41,42,43,44,45]. Authors have also studied various metal deposition techniques in the past 5 years and studied their performance to reduce delamination [46,47,48,49,50,51,52,53,54,55].

The objective of the present work is to carry out finite element analysis of commonly used coatings for micro-milling applications on high-speed steel substrates and evaluate the following.

- Objective 1: to model delamination of the coating from the substrate for micro-milling applications and find out factors on which delamination depends.
- Objective 2: to evaluate the performance of different coating materials for delamination and report the best coating material for micro-milling applications and their corresponding thickness.

The above mentioned objectives can be achieved by carrying out finite element analysis with high speed steel as a substrate with different coating materials of different thicknesses. In the present study, three-point bending was examined to simulate the practical conditions of the micro-milling tools during machining. The FEA results were validated using analytical results.

## 3. CAD Model of Coating and Substrate Design to Study Delamination (Objective 1)

### 3.1. Designing of Coating and Substrate Assembly

**Figure 2:**
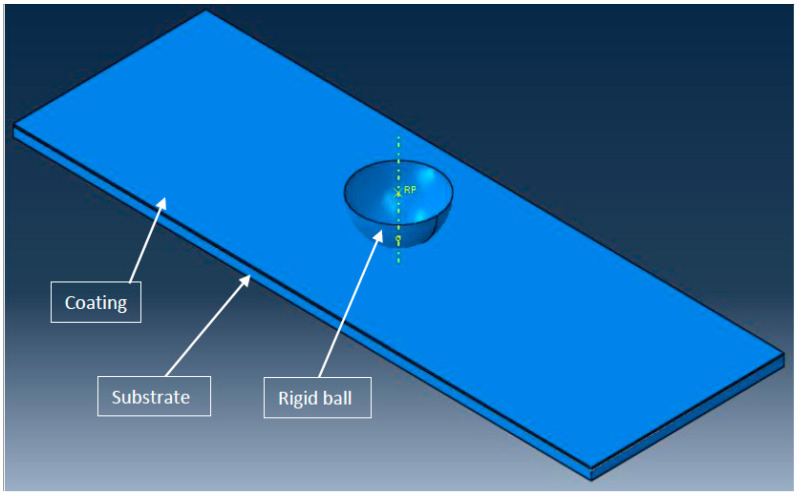
Abaqus model of coating and substrate assembly with rigid ball.

A 3D finite element model of coating and substrate assembly was developed in Abaqus to find the Hertzian normal stress when subjected to normal load of 4N, applied with the help of a rigid ball, as shown in Figure 2. To validate the FEA results, it was compared with the analytical result of Hertzian normal stress. Earlier, the ball on a flat coating–substrate assembly was used to simulate the scratch test [37]. The dimensions of the FEA model are as follows: the radius of rigid ball is 200 µm. For the TiN coating, the length is 3 mm, the width is 1 mm, and the thickness is 3 µm. For the high speed steel substrate, the length is 3 mm, the width is 1 mm, and the thickness is 50 µm. Table 1 elaborates the material properties of the coating and substrate.

### 3.2. Mechanical Properties of Different Coatings and the Substrate

The deformation behaviour of all the coating materials is considered as linear–elastic and that of the substrate is characterized as elastic–plastic, which was already mentioned earlier. The substrate material for all four coating materials was taken as high-speed steel (HSS). Material properties of different coating materials and substrate are given in Table 2 [37,43,44,45]. The following values were considered for the analysis.

### 3.3. Assumptions in the Present Study

In the present study, the following assumptions are made based on which analysis was carried out.

- The deformation behaviour of the substrate was characterized as elastic–plastic with isotropic hardening.
- The deformation behaviour of the coating materials was modelled as linear–elastic.
- The hemispherical ball was modelled as analytical rigid.
- For the surface interaction between the ball and coating material, the outer surface of the hemispherical ball was considered as the master surface and the top surface of the coating material was considered as the slave surface. This was done because the hemispherical ball was modelled as analytical rigid [37].
- For the surface interaction between the coating material and substrate, the bottom surface of the coating was considered as the master surface and the top surface of the substrate was modelled as the slave surface as the coating material was harder than the substrate.
- The interaction property for the junction of the coating and substrate was modelled as a ‘tie’ such that there was no slip, separation and penetration.
- Brick elements were taken for both the coating and substrate, and the element type was taken as the quadratic with the hybrid formulation and reduced integration. The structured type of mesh control is used.

### 3.4. Dimensioning and Boundary Conditions

**Figure 3:**
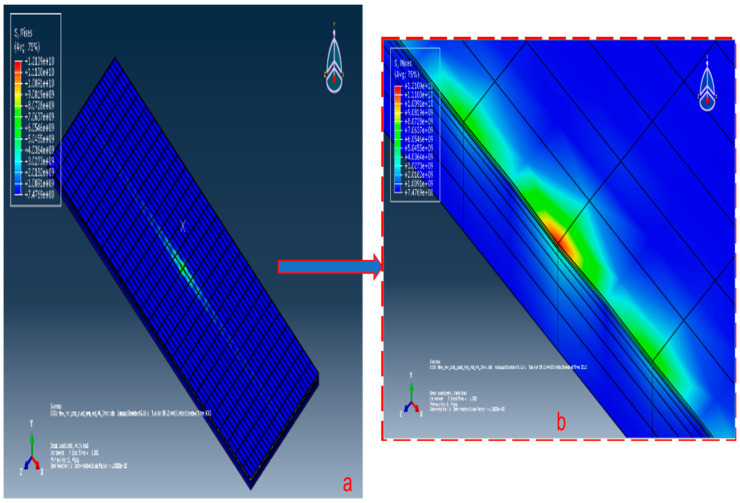
Stress contour for 4 N normal loads on coating–substrate assembly.

- Along the length and width, the coating and substrate both were divided into 26 equal parts. Along the thickness, the coating material was divided into two equal parts and the substrate was divided into five parts with single bias with the bias ratio as five. Along the length, the mesh size of the coating material and substrate was 115 µm; along the width, the mesh size of the coating material and substrate was 38 µm; along the thickness, the mesh size for the coating material was 1.5 µm; and for the substrate, the mesh size ranged from 4 µm to 19 µm. The interaction property for the junction of the coating and substrate was modelled as a ‘tie’ such that there was no slip, separation and penetration.
- The bottom of the substrate was given a rigid support and a normal load of 4 N was applied at the reference point of the rigid ball. The stress contour thus obtained is shown in Figure 3a,b.

Now the above FEA model of coating-substrate assembly is treated as simply supported beam and is subjected to a normal load of 4 N centrally throughout the width of the coating-substrate assembly and analysed for three-point bending test. From previously reported results on micro cutting tool it was found experimentally that cutting force varies between 1.5 N to 2 N. Therefore, in view of the above, for the present analysis a load of 4 N is taken considering extreme conditions. Four different coating materials are used for analysis, which are commonly used for micro-milling applications. Dimensions of each coating materials and substrate used in the FEA model are tabulated in Table 3.

### 3.5. Surface-Based Cohesive Behaviour

To simulate the delamination of the coating, the interface of different coatings and the substrate was modelled with cohesive surface in Abaqus. Surface-based cohesive behaviour was used to simulate the interface adhesion between the different coatings and substrate since the interface thickness was negligibly small. Cohesive behaviour is defined by means of the surface interaction property between the surfaces of the coating material and substrate material coming in contact with each other. The surface of the coating interacting with substrate was considered as the master surface since all the coating materials are harder than the substrate. The surface of the substrate interacting with coating was considered as the slave surface. Cohesive behaviour is defined by specifying the stiffness coefficients: Knn, Kss and Ktt for uncoupled traction–separation behaviour, where Knn represents the stiffness coefficient for the cohesive behaviour–enabled surface interaction in the normal direction and Kss and Ktt represent the stiffness coefficients for the cohesive behaviour–enabled surface interaction in the shear directions. However, it is advisable to keep the same stiffness value for the stiffness coefficients Knn, Kss and Ktt [47].

The stiffness value of the interface of the coating material and substrate is given by the relation given in Equation number (1) [48].

(1) $${\left(E/H\right)}_{i}{}^{1/2}=\frac{{\left(E/H\right)}_{s}{}^{1/2}}{1+{\left(H_{s}/H_{c}\right)}^{1/2}}+\frac{{\left(E/H\right)}_{c}{}^{1/2}}{1+{\left(H_{c}/H_{s}\right)}^{1/2}}$$

In equation number (1), ‘i’ represents the interface, ‘s’ represents the substrate, ‘c’ represents the coating, E represents the modulus of elasticity and H represents the Vickers hardness. The hardness of the interface between the coating material and substrate is assumed as the average hardness of the coating material and substrate. The interface hardness, Hi, is given by Equation number (2).

(2) $$H_{i}=\frac{H_{S}+H_{C}}{2}$$

The stiffness coefficients of the interface of the different coating–substrate assemblies Ei or Knn, Kss and Ktt are calculated by using Equation number (2) and are given in Table 4. These values are used for the analysis.

All the required inputs are fed into the FEA model of the different coating–substrate assemblies with three different coating thicknesses of 2, 3 and 4 µm with the corresponding mesh size of the coating material along the thickness as 2, 1.5 and 2 µm, respectively. All the models run for three-point bending load condition to evaluate desirable outputs such as the von Mises stress, the plastic equivalent strain and the deformation in the coating material and substrate for each case. Consolidated results of the desirable outputs of all FEA models are shown in the results section.

## 4. Analytical Calculation for Hertzian Normal Stress

**Figure 4:**
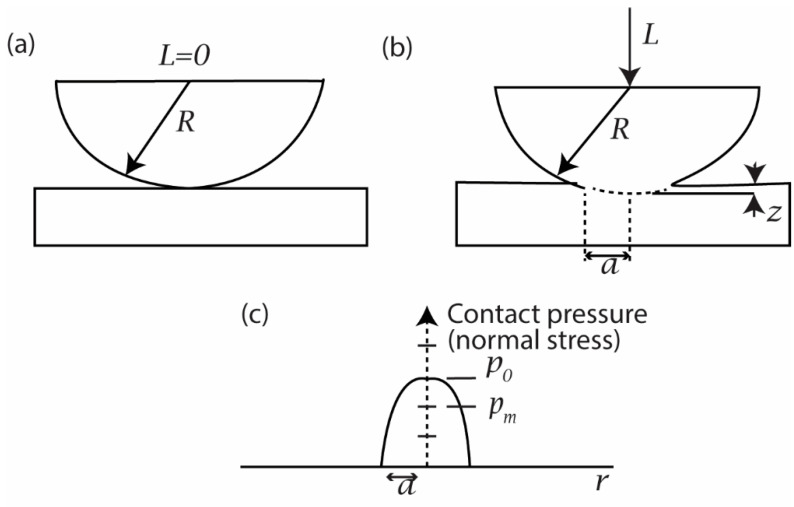
Sphere on flat-contact geometry with (**a**) no load and (**b**) load = L. (**c**) The distribution of normal stress across the contact zone of (**b**).

When a spherically shaped summit of radius R is brought into contact with a flat surface with a load L, as shown in Figure 4, the surfaces deform to create the contact zone of radius a. According to Hertz’s equations for the elastic deformation of a sphere on a flat surface, the radius of the contact zone is given by

(3) $$a={\left(\frac{3\mathrm{RL}}{4\mathrm{Ec}}\right)}^{1/3}$$

where Ec is the composite elastic modulus of the two contacting materials with the elastic modulus E_1_ and E_2_ and the Poisson’s ratio ν_1_ and ν_2,_ respectively. The value of Ec is given by the relation as given below.

(4) $$\frac{1}{E_{c}}=\frac{1-\nu_{1}{}^{2}}{E_{1}}+\frac{1-\nu_{2}{}^{2}}{E_{2}}$$

For this geometry, the real area of contact A is given by

(5) $$A={\pi a}^{2}$$

(6) $$A=\pi {\left(\frac{3\mathrm{RL}}{4\mathrm{Ec}}\right)}^{2/3}$$

The mean normal stress, pm, is given by

(7) $$p_{m}=\frac{L}{A}$$

or,

(8) $$p_{m}=\frac{1}{\pi }{\left(\frac{4E_{c}}{3R}\right)}^{2/3}L^{1/3}$$

The maximum normal stress, po, is given by

(9) $$\mathrm{po}=\frac{3p_{m}}{2}$$

When a rigid ball of radius R = 200 µm and elastic modulus E_1_ = ∞ is pressed with a normal load of 4 N against a flat plate of TiN with elastic modulus E_2_ = 300 GPa and Poisson’s ratio ν = 0.22, then the maximum normal stress obtained, po, is given in Table 5.

The above loading conditions of a load of 4 N through a rigid ball pressed against a flat plate of TiN is simulated in Abaqus 6.11–1. The maximum normal stress developed in FEA model was 12,109 MPa. The variation of the FEA result from the analytical result, i.e., 12,435.97 MPa, is 2.63%, which is acceptable. This confirms that the FEA model of the coating–substrate assembly is acceptable.

**Figure 5:**
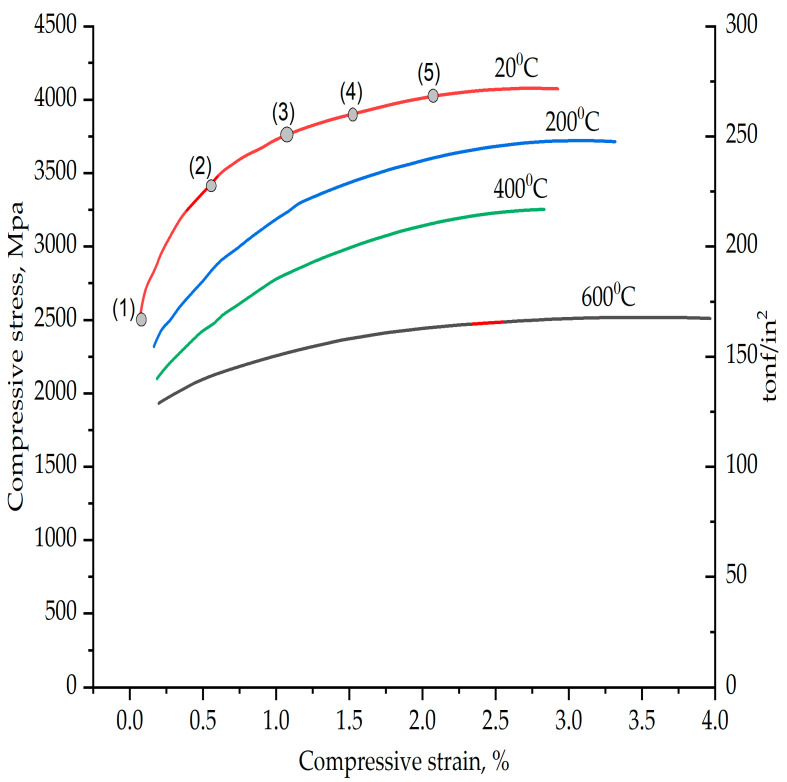
Compressive stress v/s compressive strain curve of high-speed steel [46].

The plastic strain curve of the high-speed steel material used as a substrate is shown in Figure 5 [46]. With reference to Table 6, for compressive strain, v/s compressive stress values of the HSS five data point values are extracted for the plastic behaviour of high-speed steel corresponding to a 20 °C curve, as shown in Figure 5. This plastic behaviour data were added into the Abaqus 6.11–1 software as the plastic behaviour of the high-speed steel.

## 5. Results and Discussion: Performance Evaluation of Different Coating Materials in Delamination (Objective 2)

**Figure 6:**
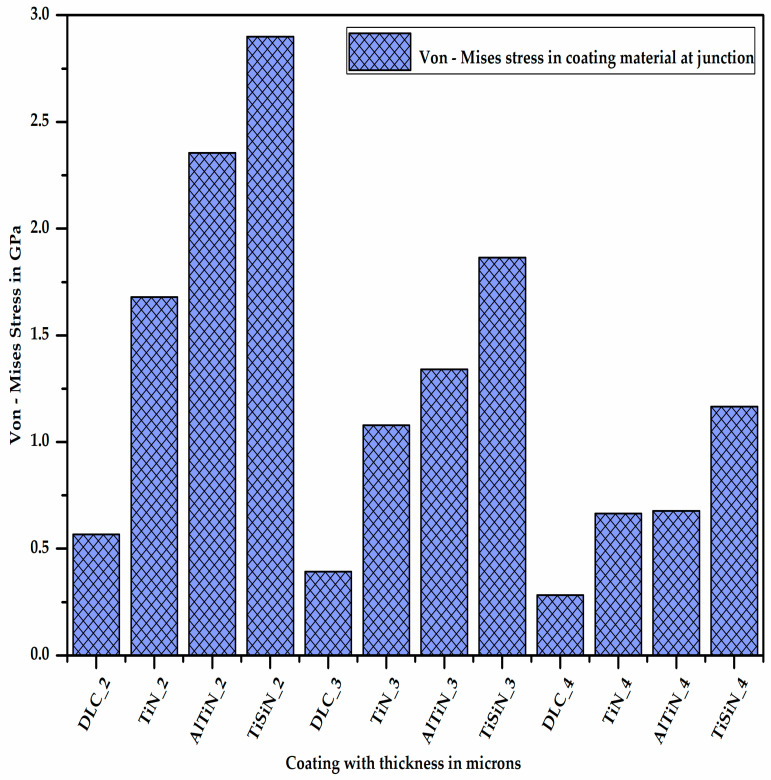
Von Mises stress in coating material at junction.

All the FEA models of the different coating–substrate assemblies with three different coating thicknesses of 2, 3 and 4 µm were run for the three-point bending load condition to evaluate the von Mises stress, the plastic equivalent strain and the deformation in the coating materials and substrate. Contour plots of the desirable outputs are given in the Appendix A. The consolidated results of the desirable outputs of all FEA models are shown in this section. Figure 6 shows the maximum von Mises stress acting on the coating material at the junction of the coating and substrate, situated below the loading region, where AlTiN_3 represents the coating of aluminium titanium nitride material of 3 micron thickness.

**Figure 7:**
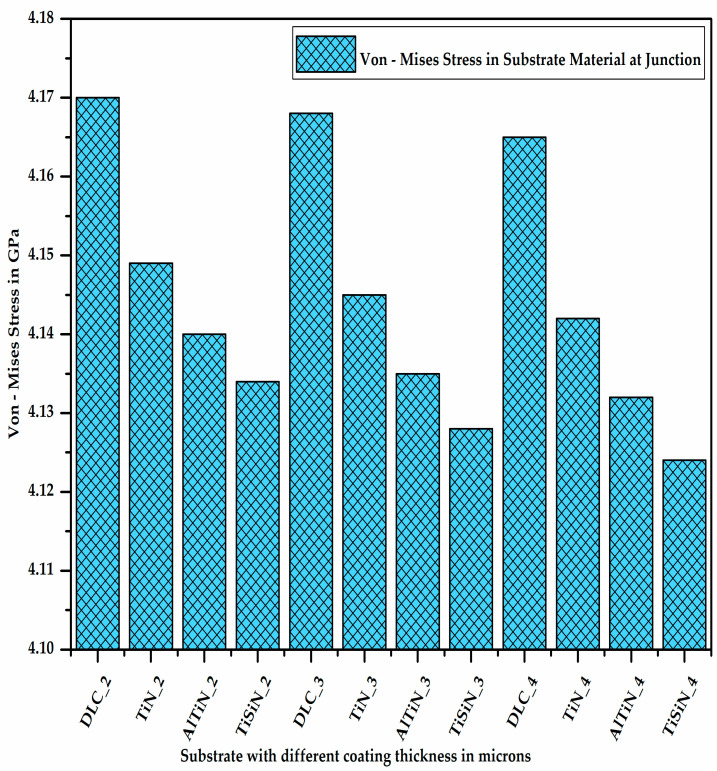
Von Mises stress in substrate material at junction.

It is quite clear from Figure 6 that as the thickness of the coating material increases, the stress developed on the coating material decreases since the section modulus of the coating increases. Additionally, for a given thickness of coating material, the stress developed in the diamond-like carbon coating material is minimum (DLC_4) and that in the titanium silicon nitride coating material is maximum (TiSiN_4), which means the stress bearing capacity of the DLC coating is minimum and that of the TiSiN is maximum. Figure 7 shows the maximum von Mises stress acting on the substrate at the junction of the coating material and substrate, situated below the loading region with different coating materials of different thicknesses.

It can be observed from Figure 7 that the stress developed on the substrate material decreases with the increase in the coating thickness due to the increase in the section modulus of the coating–substrate assembly. Additionally, for a given thickness of the coating material, the stress developed on the substrate is maximum in the case of the DLC coating and minimum in the case of the TiSiN coating. This shows that the TiSiN coating material bears most of the stress developed due to the application of the load preventing the substrate from experiencing high stress, unlike the other coating materials.

**Figure 8:**
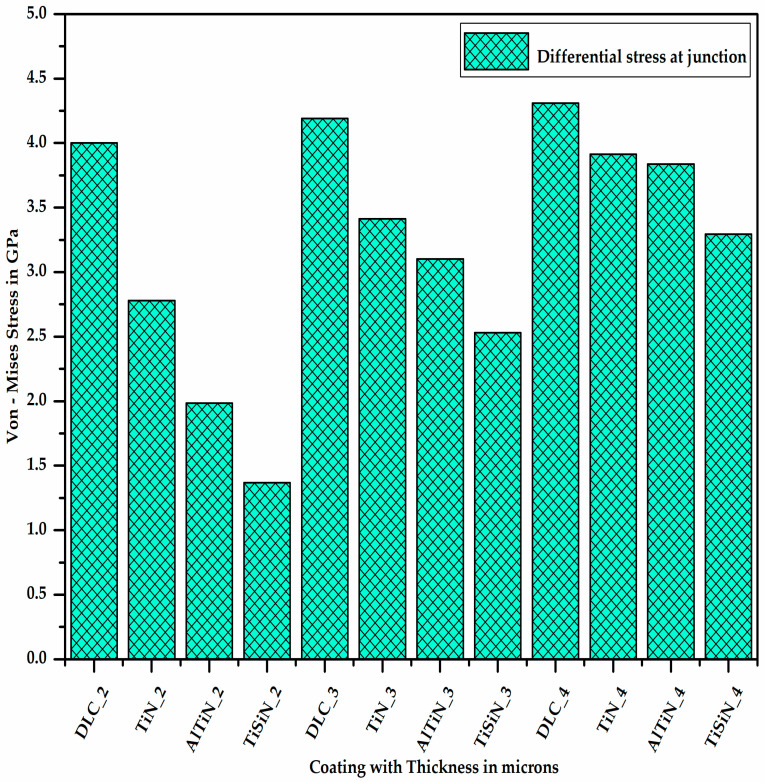
Differential stress at junction of coating material and substrate.

Figure 8 shows the differential stress at the junction of the coating and substrate, which is nothing but the difference of the stresses experienced by the coating material and substrate. It is clear from Figure 8 that for a given thickness of the coating material, the differential stress between the coating material and substrate at the junction is maximum for DLC and minimum for TiSiN. As the thickness of the coating material increases, the differential stress at the junction of the coating material and substrate increases.

**Figure 9:**
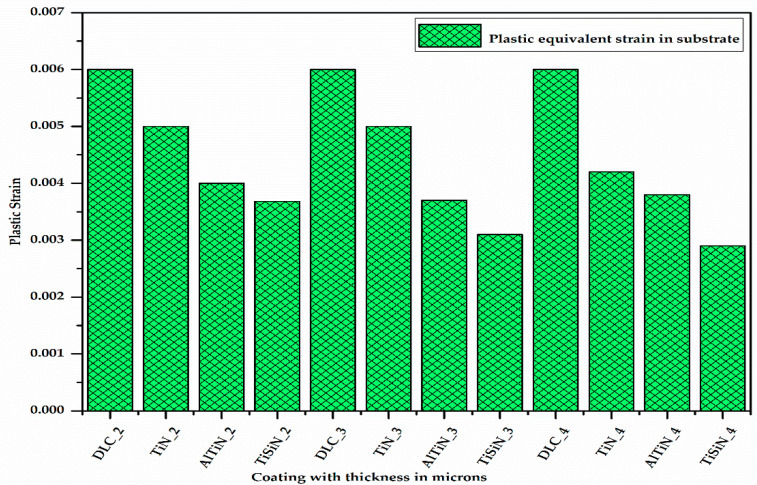
Plastic equivalent strain in substrate with different coating materials of varying thicknesses.

The differential stress at the junction of the substrate and coating material causes some plastic strain at the substrate surface due to the difference in the hardness of the coating material and substrate material. Figure 9 shows the plastic equivalent strain developed on the substrate with different coating materials of different thicknesses. It can be seen from Figure 9 that for a given coating material, as the thickness of the coating increases, the plastic equivalent strain in the substrate decreases as the stress developed in the substrate decreases. Additionally, for a given thickness of the coating, the plastic equivalent strain in the substrate is maximum for the DLC coating and minimum for the TiSiN coating.

**Figure 10:**
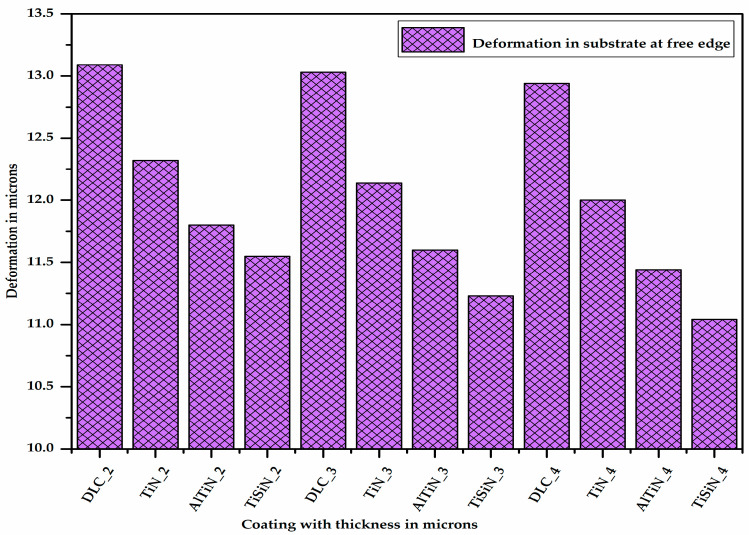
Deformation in substrate at free edge with different coating materials of varying thicknesses.

Figure 10 shows the deformation in the substrate at one of the corners at the free edge having coatings of different materials with varying thicknesses. It is very clear from Figure 10 that as the thickness of the coating increases, the deformation in the substrate decreases. It can be also seen (Figure 13) that for a given thickness of coating, the deformation in the substrate is maximum for the DLC coating and minimum for the TiSiN coating.

**Figure 11:**
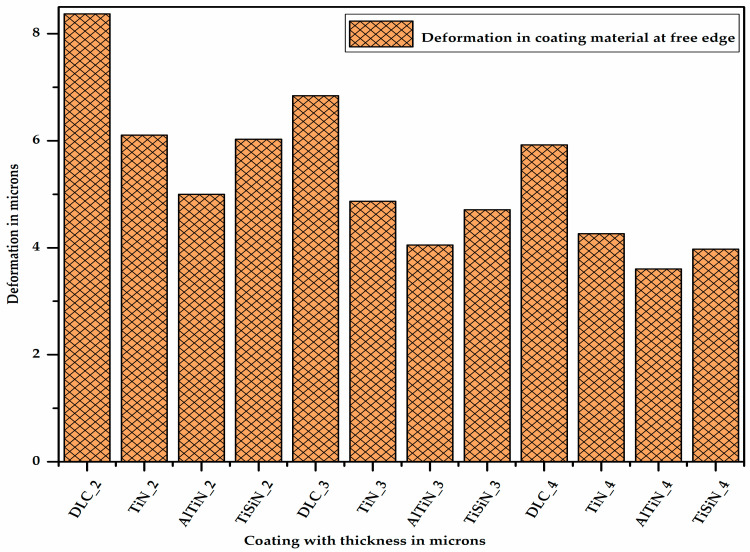
Deformation in different coating materials of varying thicknesses.

Figure 11 shows the deformation in the different coating materials of varying thicknesses at the junction of the coating material and the substrate at one of the corners of the free edge. It can be observed from Figure 13 that for a given thickness of coating, the deformation is maximum for the DLC coating and minimum for the AlTiN coating. This is because the deformation is inversely proportional to the elastic modulus. As the thickness of coating material increases, its deformation decreases due to the increase in its section modulus.

**Figure 12:**
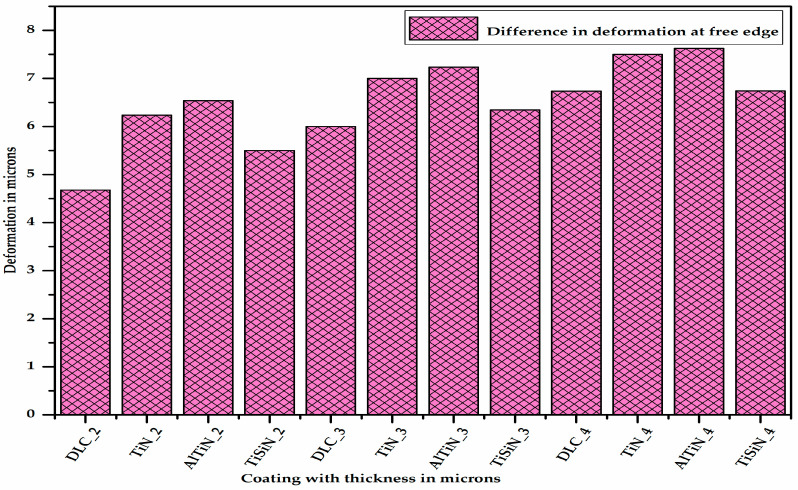
Difference in deformation between different coating materials of varying thicknesses and substrate.

**Figure 13:**
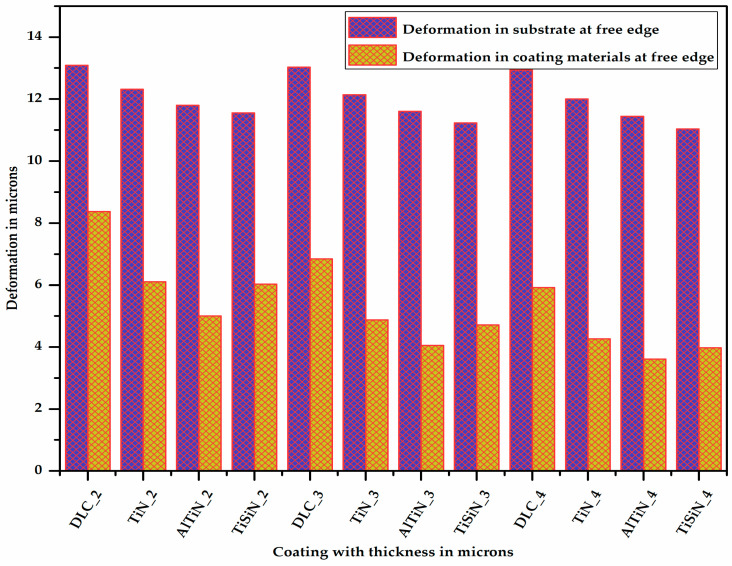
Deformation comparison between substrate and coating materials at free edge.

Figure 12 shows the difference in the deformation between the different coating materials of varying thicknesses and the substrate located at one of the corners at the free edge, thereby showing the extent of the delamination. It can be said from Figure 12 that for a given thickness of coating, the difference in the deformation between the coating material and substrate is the maximum for the AlTiN coating and minimum for the DLC coating. From Figure 13 it can be concluded that as the thickness of coating material increases, the difference in the deformation between the coating material and substrate, i.e., delamination, increases.

Because of the non-availability of FEA results in the literature, the results of the present study were not compared. However, it is clear from the result that aluminium titanium nitride coating material performs better than titanium nitride, which was reported earlier in the literature.

## 6. Conclusions

From the different consolidated results of the desired outputs, the following conclusions can be made:

1. Using a 3D modelling approach, the coating and substrate assembly was modelled and studied for delamination. Various factors were evaluated which impacted the delamination in micro-milling.
2. On the basis of the plastic equivalent strain occurring in the substrate material, the TiSiN and AlTiN coatings are the best since the plastic equivalent strain occurring in the substrate with the TiSiN and AlTiN coating materials is less (Appendix A). Between these two, the TiSiN coating shows the least plastic equivalent strain in the substrate, which serves the purpose of protecting the substrate from plastic deformation. The plastic equivalent strain in the substrate decreases with increase in the thickness of the coating material.
3. On the basis of the difference in deformation, i.e., delamination, the TiSiN coating is better than the AlTiN coating since delamination in the coating–substrate assembly with the TiSiN coating is less than that of the AlTiN coating. The delamination of the coating material from the substrate increases with increase in the thickness of the coating material.
4. By combining the plastic equivalent strain in the substrate and the delamination of the coating from the substrate, we can conclude that the TiSiN coating of 3 micron thickness is the most optimum coating thickness for micro-milling applications. These results fulfill the requirements of objectives 1 and 2.
5. The higher the interface stiffness coefficient of the coating–substrate assembly is, the less the delamination of the coating material from the substrate is.
6. The delamination depends on the Young’s modulus and the hardness of both the coating material and substrate.

**Suggestions for future work:** In order to validate the FEA results obtained, experimental studies are essential. Therefore, these values will help in comparing the FEA results. An experimental setup is required to see the delamination process. Different coatings on the substrate are required to be generated to conduct the experiment. The mechanical properties of the coatings need to be evaluated. SEM images can be taken to evaluate the extent of the delamination occurring on different coating materials.

## Figures and Tables

**Table 1:** Mechanical properties of coating and substrate.

Sl No.	Type	Material	Young’s Modulus	Poisson’s Ratio
1	Coating	TiN	300 GPa	0.22
2	Substrate	High-speed steel	200 GPa	0.29

**Table 2:** Mechanical properties of different coating materials and substrate.

S. No.	Type	Material	Young’s Modulus (GPa)	Hardness (GPa)	Poisson’s Ratio
1	Coating	TiN	300	27	0.22
2	Coating	DLC	70	10.5	0.22
3	Coating	AlTiN	560	35	0.22
4	Coating	TiSiN	510	56	0.20
5	Substrate	HSS	200	7.5	0.29

**Table 3:** Dimensions of different coating materials and substrate in FEA models.

Sl No.	Type	Material	Length	Width	Thickness
1	Coating	Titanium Nitride	3 mm	1 mm	2, 3 and 4 µm
2	Coating	Diamond-like Carbon	3 mm	1 mm	2, 3 and 4 µm
3	Coating	Aluminium Titanium Nitride	3 mm	1 mm	2, 3 and 4 µm
4	Coating	Titanium Silicon Nitride	3 mm	1 mm	2, 3 and 4 µm
5	Substrate	High-speed Steel	3 mm	1 mm	50 µm

**Table 4:** Interface stiffness coefficients of different coating–substrate assemblies.

Sl No.	Coating–Substrate Assembly	Ec (GPa)	Hc (GPa)	Es (GPa)	Hs (GPa)	Hi = (Hs + Hc)/2 (GPa)	Ei or Knn, Kss, Ktt (GPa)
1	TiN-HSS	300	27	200	7.5	17.25	357
2	DLC-HSS	70	10.5	200	7.5	9	143
3	AlTiN-HSS	560	35	200	7.5	21.25	487
4	TiSiN-HSS	510	56	200	7.5	31.75	665

**Table 5:** Value of composite elastic modulus and normal stresses.

S. N.	Particulars	Symbol	Equation Number Used	Value
1	Composite elastic modulus	Ec	3.2	315.26 GPa
2	Mean normal stress	pm	3.6	8290.64 MPa
3	Maximum normal stress	po	3.7	12,435.97 MPa

**Table 6:** Plastic behaviour of HSS.

Data Point	Compressive Stress (MPa)	Compressive Strain
1	2450	0
2	3470	0.005
3	3750	0.01
4	3820	0.015
5	4000	0.02

**Figure A1:**
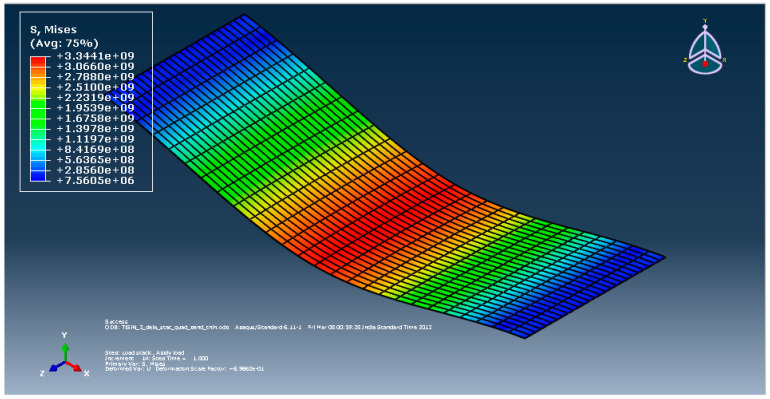
Von Mises stress contour plot of TiSiN coating with 3 µm thickness.

**Figure A2:**
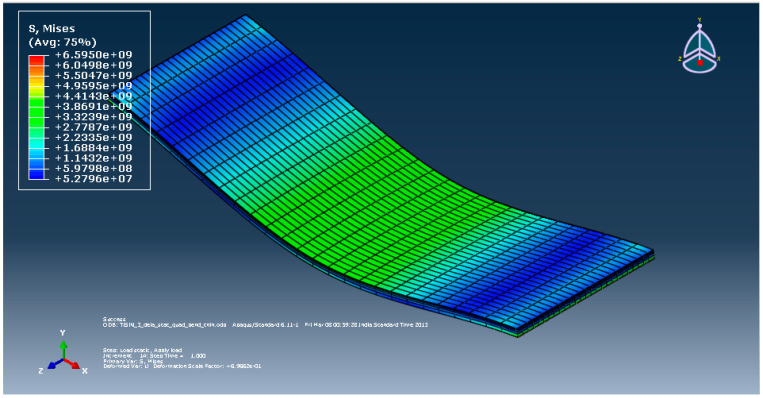
Von Mises stress contour plot of HSS substrate.

**Figure A3:**
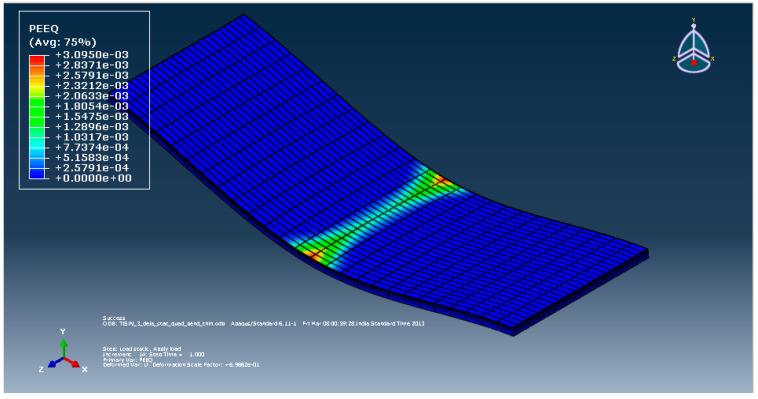
Plastic equivalent strain contour plot of HSS substrate with TiSiN coating thickness of 3 µm.

**Figure A4:**
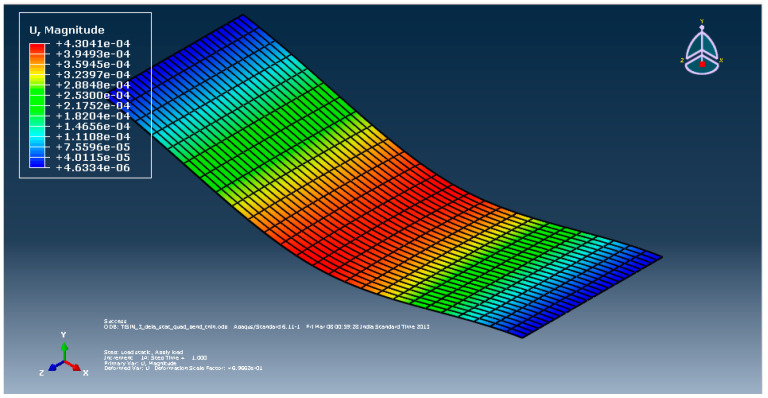
Deformation contour plot of TiSiN coating with 3 µm thickness.

**Figure A5:**
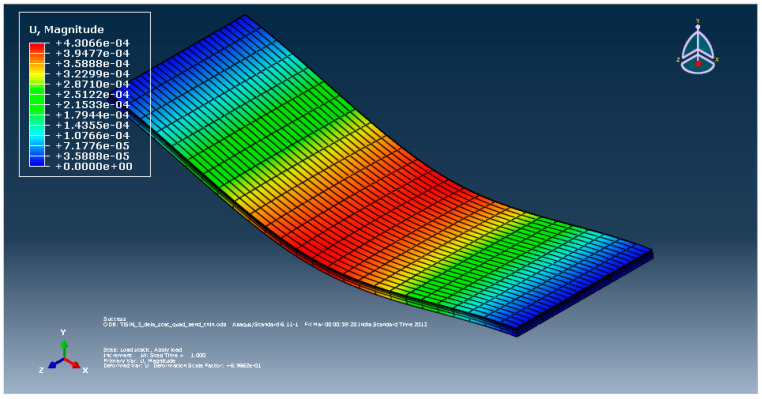
Deformation contour plot of HSS substrate with 3 µm coating thickness of TiSiN.

## Appendix A

The contour plots of the desirable outputs of the FEA model with titanium silicon nitride coating of 3 µm thickness over high-speed steel substrate are as follows.

## References

[1] Aramcharoen A., Mativenga P.T., Yang S., Cooke K.E., Teer D.G. (2008). Evaluation and selection of hard coatings for micro milling of hardened tool steel. Int. J. Mach. Tools Manuf..

[2] Dornfeld D., Min S., Takeuchi Y. (2006). Recent advances in mechanical micromachining. CIRP Ann.—Manuf. Technol..

[3] Masuzawa T. (2000). State of the art of micromachining. CIRP Ann.—Manuf. Technol..

[4] Weule H., Huntrup V., Tritschler H. (2001). Micro-cutting of steel to meet new requirements in miniaturization. CIRP Ann.—Manuf. Technol..

[5] Liu X., de Vor R.E., Kapoor S.G., Ehmann K.F. (2004). The mechanics of machining at the microscale: Assessment of the current state of the science. J. Manuf. Sci. Eng..

[6] Min S., Sangermann H., Mertens C., Dornfeld D. (2008). A study on initial contact detection for precision micro-mold and surface generation of vertical side walls in micromachining. CIRP Ann.—Manuf. Technol..

[7] Masuzawa T., Toenshoff H.K. (1997). Three-dimensional micromachining by machine tools. CIRP Ann. —Manuf. Technol..

[8] Bissacco G., Hansen H.N., de Chiffre L. (2005). Micromilling of hardened tool steel for mould making applications. J. Mater. Process. Technol..

[9] Bissacco G., Hansen H.N., de Chiffre L. (2006). Size effects on surface generation in micro milling of hardened tool steel. CIRP Ann.—Manuf. Technol..

[10] Li P., Oosterling J.A.J., Hoogstrate A.M. Performance evaluation of micromilling of hardened tool steel. Proceedings of the ICOMM.

[11] Aramcharoen A., Mativenga P.T., Yang S. The effect of AlCrTiN coatings on product quality in micro-milling of 45 HRC hardened H13 die steel. Proceedings of the 35th International Matador Conference.

[12] Maekawa K., Obikawa T., Yamane Y., Childs T.H.C. (2000). Metal Machining—Theory and Applications.

[13] Kobayashi A. (2005). The features and application of UPC nano-micro forming tools. Ind. Diam. Rev..

[14] Kim C.J., Bono M., Ni J. (2002). Experimental Analysis of Chip Formation in Micro-Milling.

[15] Jun M.B.G., DeVor R.E., Kapoor S.G. (2006). Investigation of the dynamics of microend milling-Part II: Model validation and interpretation. J. Manuf. Sci. Eng..

[16] Torres C.D., Heaney P.J., Sumant A.V., Hamilton M.A., Carpick R.W., Pfefferkorn F.E. (2009). Analyzing the performance of diamond-coated micro end mills. Int. J. Mach. Tools Manuf..

[17] Ozel T., Lui X., Dhanorker A. Modelling and simulation of micro-milling process. Proceedings of the 4th International Conference and Exhibition on Design and Production of Machines and Dies/Molds.

[18] Tansel I., Rodriguez O., Trujillo M., Paz E., Li W. (1998). Micro-end-milling-I. Wear and breakage. Int. J. Mach. Tools Manuf..

[19] Baharudin B.T.H.T., Dimou N., Hon K.K.B. Tool wear behaviour of micro-tools in high speed CNC machining. Proceedings of the 34th International MATADOR Conference.

[20] Uhlmann E., Schauer K. (2005). Dynamic load and strain analysis for the optimization of micro end mills. CIRP Ann.—Manuf. Technol..

[21] Rahman M., Kumar A.S., Prakash J.R.S. (2001). Micro milling of pure copper. J. Mater. Process. Technol..

[22] Bissacco G., Hansen H.N., Chiffre L.D. Wear of micro end mills. Proceedings of the 5th EUSPEN International Conference.

[23] Zaman M.T., Kumar A.S., Rahman M., Sreeram S. (2006). A three-dimension analytical cutting force model for micro end milling operation. Int. J. Mach. Tools Manuf..

[24] Uriarte L., Zatarian M., Albizuri J., Lacalle L.N.L.d., Lamikiz A. Effect of the tool wear in micro-milling cutting forces. Proceedings of the 2nd International Conference on High Performance Cutting.

[25] Takacs M., Vero B., Meszaros I. (2003). Micromilling of metallic materials. J. Mater. Process. Technol..

[26] Tansel I.N., Arkan T.T., Bao W.Y., Mahendrakar N., Shisler B., Smith D., McCool M. (2000). Tool wear estimation in micro-machining, part II: Neural network-based periodic inspector for non-metals. Int. J. Mach. Tools Manuf..

[27] Schmidt J., Tritschler H. (2004). Micro cutting of steel. Microsyst. Technol..

[28] Uhlmann E., Piltz S., Schauer K. (2005). Micro milling of sintered tungsten-copper composite materials. J. Mater. Process. Technol..

[29] Vogler M.P., DeVor R.E., Kapoor S.G. (2004). On the modeling and analysis of machining performance in micro-endmilling, Part I: Surface generation. J. Manuf. Sci. Eng..

[30] Weinert K., Petzoldt V. (2008). Machining NiTi micro-parts by micro-milling. Mater. Sci. Eng. A.

[31] Aramcharoen A., Mativenga P.T., Yang S. The contribution of CrTiAlN coatings in micro milling of hardened die steel. Proceedings of the 40th CIRP International Seminar on Manufacturing Systems.

[32] Bin Saedon J. (2011). Micromilling of Hardened (62 HRC) AISI D2 Cold Work Tool Steel. Ph.D. Thesis.

[33] Dobrzanski L.A., Golombek K., Kopac J., Sokovic M. (2004). Effect of depositing the hard surface coatings on properties of the selected cemented carbides and tool cermets. J. Mater. Process. Technol..

[34] Talib R.J., Ariff H.M., Fazira M.F. (2011). Machining performance and wear mechanism of TiAlN-coated insert. Int. J. Mech. Mater. Eng. (IJMME).

[35] Kumar M. (2011). Laser Assisted Micro Milling of Hard Materials. Ph.D. Thesis.

[36] Scherzer M., Glaser H. (2002). Mechanical Modelling of Failure Patterns in TiN-Coatings Loaded by Homogenous Stresses of a Bending Specimen. Int. J. Fract..

[37] Holmberg K., Laukkanen A., Ronkainen H., Wallin K., Varjus S., Koskinen J. (2006). Tribological contact analysis of a rigid ball sliding on a hard coated surface. Part II: Material deformations, influence of coating thickness and Young’s modulus. Surf. Coat. Technol..

[38] Holmberg K., Laukkanen A., Ronkainen H., Wallin K., Hogmark S., Jacobson S., Wiklund U., Souza R.M., Stahle P. (2009). Residual stresses in TiN, DLC and MoS2 coated surfaces with regard to their tribological fracture behaviour. Wear.

[39] Wu T., Cheng K. (2013). Micro milling performance assessment of diamond-like carbon coatings on a micro-end mill. Proc. Inst. Mech. Eng. Part J J. Eng. Tribol..

[40] Ucun I., Aslantas K., Bedir F. (2013). An experimental investigation of the effect of coating material on tool wear in micro milling of Inconel 718 super alloy. Wear.

[41] Tao Wu T. (2012). Tooling Performance in Micro Milling: Modelling, Simulation and Experimental Study. Ph.D. Thesis.

[42] Cselle T., Coddet O., Galamand C., Holubar P., Jilek M., Jilek J., Luemkemann A., Morstein M. (2009). Triple coatings—New Generation of PVD-Coatings for Cutting Tools. J. Mach. Manuf..

[43] Lille H., Koo J., Gregor A., Ryabchikov A., Sergejev F., Traksmaa R., Kulu P. (2011). Comparison of Curvature and X-Ray Methods for Measuring of Residual Stresses in Hard PVD Coatings. Mater. Sci. Forum.

[44] Ahmed M.S., Munroe P., Jiang Z.T., Rickard W., Xie Z. (2011). Corrosion behaviour of nano-composite TiSiN coatings on steel substrates. Corros. Sci..

[45] Chang Y.Y., Yang S.J., Wang D.Y. (2006). Structural and mechanical properties of AlTiN/CrN coatings synthesized by a cathodic-arc deposition process. Surf. Coat. Technol..

[46] Trent E.M., Paul K. (2000). Metal Cutting.

[47] Surface Based Cohesive Behaviour 35.1.10.

[48] Lesage J., Chicot D. (2002). Role of residual stresses on interface toughness of thermally sprayed coatings. Thin Solid Films.

[49] Zhang J., Cho Y., Kim J., Malikov A.K., Kim Y.H., Yi J.-H., Li W. (2021). Non-Destructive Evaluation of Coating Thickness Using Water Immersion Ultrasonic Testing. Coatings.

[50] Šporin J., Mrvar P., Janc B., Vukelić Ž. (2021). Expression of the Self-Sharpening Mechanism of a Roller Cone Bit during Wear Due to the Influence of the Erosion Protection Carbide Coating. Coatings.

[51] Dettlaff A., Brodowski M., Kowalski M., Stranak V., Prysiazhnyi V., Klugmann-Radziemska E., Ryl J., Bogdanowicz R. (2021). Highly oriented zirconium nitride and oxynitride coatings deposited via high-power impulse magnetron sputtering: Crystal-facet-driven corrosion behavior in domestic wastewater. Adv. Eng. Mater..

[52] Zheng L., Chen W., Huo D. (2020). Investigation on the Tool Wear Suppression Mechanism in Non-Resonant Vibration-Assisted Micro Milling. Micromachines.

[53] Kino H., Imada T., Ogawa K., Nakagawa H., Kojima H. (2020). An Experimental Investigation on Micro End Milling with High-Speed Up Cut Milling for Hardened Die Steel. Materials.

[54] Grigoriev S.N., Migranov M.S., Melnik Y.A., Okunkova A.A., Fedorov S.V., Gurin V.D., Volosova M.A. (2021). Application of Adaptive Materials and Coatings to Increase Cutting Tool Performance: Efficiency in the Case of Composite Powder High Speed Steel. Coatings.

[55] Asghar O., Lou L.Y., Yasir M., Li C.J., Li C.X. (2020). Enhanced Tribological Properties of LA43M Magnesium Alloy by Ni60 Coating via Ultra-High-Speed Laser Cladding. Coatings.

